# Science Christianity’s Handmaid

**Published:** 1877-08

**Authors:** 


### SCIENCE CHRISTIANITY'S HANDMAID.

Science is the knowledge of facts and principles; art is their application to the utilities of life. It is a scientific fact that the magnet points unerringly to the pole; by the application of this fact the mariner confidently navigates the trackless seas. On the scientific principle that nature abhors a vacuum, at least as high as thirty-two feet, the common water-pump is constructed. Science is truth itself as to all material things, as religion is truth itself as to the moral and spiritual world; hence, science and religion must sustain each other, for truth stands by truth throughout the boundless empire of Omnipotence!

If then Christianity is a truth, science must sustain it wherever and whenever they come in contact, on whatever field they meet; and Christianity being founded on the Bible, every Bible assertion will be corroborated by science whenever science speaks at all in reference to that assertion. In other words, as new facts are eliminated in the world's daily history, they can never fail to corroborate the assertions and assumptions of the Bible whenever they bear testimony to a common point. If therefore, the Bible should assert a fact of which there is no historical record, no confirmatory testimony in the world's history, the Christian has every thing to hope, and nothing to fear, from investigation, from research, from actual discovery; hence, a true system of religion must become firmer and broader and deeper in its foundations, and towering the higher too as true knowledge advances. Hence also, true religion finds its interest in promoting learning, in fostering educational plans, in encouraging laborious investigation and brave research; for it looks to the light of truth and knowledge, and glories in it, as the flower looks upwards and basks in the light of the sun, while false systems hate the light, come not to it; they fear education and elevation and liberty, for their highest hope is in the darkness of ignorance, and in the chains of despotism.

Investigation, discovery, demonstration, these are the legitimate fields of science, these are its proper work, and in proportion to the acquisitions made by them throughout the world, will the Scriptures be confirmed; and by the mouth of these "two witnesses," science and the Bible, will true religion stand the stronger and the firmer with each real discovery. Hence, true religion is the foster brother of education, elevation, and research, and that system which cherishes ignorance and represses thought, may be known thereby to be false in its foundations the world over.

There is no employment of the present time more deeply interesting than that of collecting some of the more remarkable discoveries of later years, those of Layard, Rawlinson, and others, and comparing their testimonies with the literal expressions of the Scriptures made three thousand years ago. Their unity of idea is amazing! *Some* Old Testament statements have, as yet, found no confirmation in any human record; others were so palpably at variance with ascertained facts a few ages later, that some of the most enthusiastic believers in its divine authenticity were dumb under their announcement, and in trembling hopefulness could only say, "Wait." Centuries passed away and yet it was "Wait." Millenniums were numbered with a bygone eternity, and they were waiting still; but within months, in number not large, the triumphant shout of victory comes through the air, crossing oceans, and traversing continents From emboweled pyramids, from exhumed cities, from inscriptions hidden from the sun-light for one and two and three thousand years agone, comes the glad pean: "The Bible is true, and its author is God!"

Centuries before the Christian era, the mournful prophet declared that the stars of heaven could not be numbered; not in the unimpressive words of a mere announcement, but in the confident manner of one who feels that it is impossible to call the fact in question—"As the host of heaven can not be numbered." The people of his time looked out upon the sky of night and contemplated with wonder, what seemed to them an innumerable host of twinkling points. But they were not innumerable. Astronomy has counted and localized every one of them, and there are but about three thousand; the human eye can see no more, not another star; but the seer had said they could not be numbered!

One day during a past century, a German apprentice-boy was amusing himself in melting glass over a flame, when the idea of the telescope flashed across his mind, subsequently revealing the fact that there are more than three thousand stars; that in our own system there are millions; that there are a hundred millions of them yet to be located; and as science and art are constructing telescopes of wider range, not only other stars, but other systems of stars are coming up from the deep depths of the midnight sky; so that up to this hour, the systems have not been numbered, while each of them counts its multitude of millions of stars. Said not Jeremiah well: "As the host of heaven can not be numbered?"

Nearly a thousand years before the Christian era, Jonah said that it was a three days' journey to compass the city of Nineveh; a statement which might well challenge the admission of the most credulous, when it is remembered that London, the largest city in the world, with its two and a half million of inhabitants, covers only seventy-six thousand acres of land, and can be easily walked around in a day; and that a city, three times its size and population ever existed, is simply an absurdity. But the news has come to us that Layard has himself made the circuit of ancient Nineveh on the eastern bank of the Tigris, and that the "three days' journey" of the prophet is still required to compass its ruins, and it was of its physical dimensions, and not of its numerical population, that Jonah spoke.

Whole pages would be required to comprise the literal confirmations of incidental Scripture allusions, which the discoveries of Champollion, and Abbott, and Layard, and Rawlinson, and Gliddon, and others, have made in their exhumation and interpretation of records on clay and stone and brass and imperishable adamant; of records preserved for two thousand years in entombed cities, traced on papyrus, stamped on coins, engraved in the solid rock, or sketched on palace wall and fallen column, on its capital and its base, on temple sill and lintel, never in a case falsifying a Scripture record, never in a case failing to corroborate when testifying to the same point.

As science then confirms true religion, it is to the highest interest of the latter to foster science, having nothing to fear, and much to hope from its development. For the encouragement which Christianity has given learning and art and science, behold the return in a single department! Three hundred years ago, copies of the Bible had to be made with the pen, while those who could read and write were so few, that not one of them could be spared by the state, so that if a traitor or a murderer could read and write, the gallows lost its due, and the bullet failed of its mark, because the "benefit of clergy," the "benefit," the advantage of being able to read, was, that he should go free. Then, it required a laboring man's pinching savings for a good lifetime to purchase a Bible; but now, by virtue of what has been done by such sons of science and art as Fulton and Watt and Morse and Bauer and Hoe and others like them, the price of a Bible can be easily earned in a single day, and these men are thus, in a sense, missionaries of the cross, with Martin and Buchanan and Judson and Heber and their brother worthies, while scientific books and papers are their "sermons" and their "tracts," the legitimate use of which is the advancement of a high form of civilization, and it is there where religion shines the brightest, and shows its greatest power. Thus is it that science and art and true Christianity go hand in hand; triple brothers are they, mutually aiding each other, the advance of one being promoted by the prosperity of the others, and as they progress man is elevated, society is purified, and the world made free. Christianity cherishes learning; learning establishes Christianity. The head and the heart are cultivated together, side by side they grow, the one in purity, the other in power; and fast friends will they be until time's ending, making on the earth meanwhile, gardens out of every desert, scattering flowers where only thorns grew before, and clearing away from every physical and moral waste the blots and blurs which mar the beauty of the material and moral world.

